# Eccrine porocarcinoma arising from the knee

**DOI:** 10.1002/ccr3.1319

**Published:** 2018-01-04

**Authors:** Kenji Gonda, Yuichi Hatakeyama, Yuichi Rokkaku

**Affiliations:** ^1^ Department of Surgery Japan Community Health Care Organization Nihonmatsu Hospital Nihonmatsu Fukushima Japan

**Keywords:** Eccrine porocarcinoma, knee mass

## Abstract

Enlarged eccrine porocarcinoma of the knee was encountered as a hemorrhagic bulky tumor. After controlling bleeding with Mohs’ paste, local excision of the lesion was the mainstay of treatment. Pathological examination revealed poroid cells, cuticle cells, and prickle cells cancer components, suggesting that malignancy must be excluded by resection.

## Introduction

Eccrine porocarcinoma usually arises in elderly patients, frequently in the extremities. We encountered a tumor originating from the knee that enlarged and was not resected easily, due to the hemorrhagic, bulky nature of the tumor. In most cases, elderly patients do not seek medical treatment and different tissue organization within the tumor is mixed.

## Case Presentation

**Figure 1:**
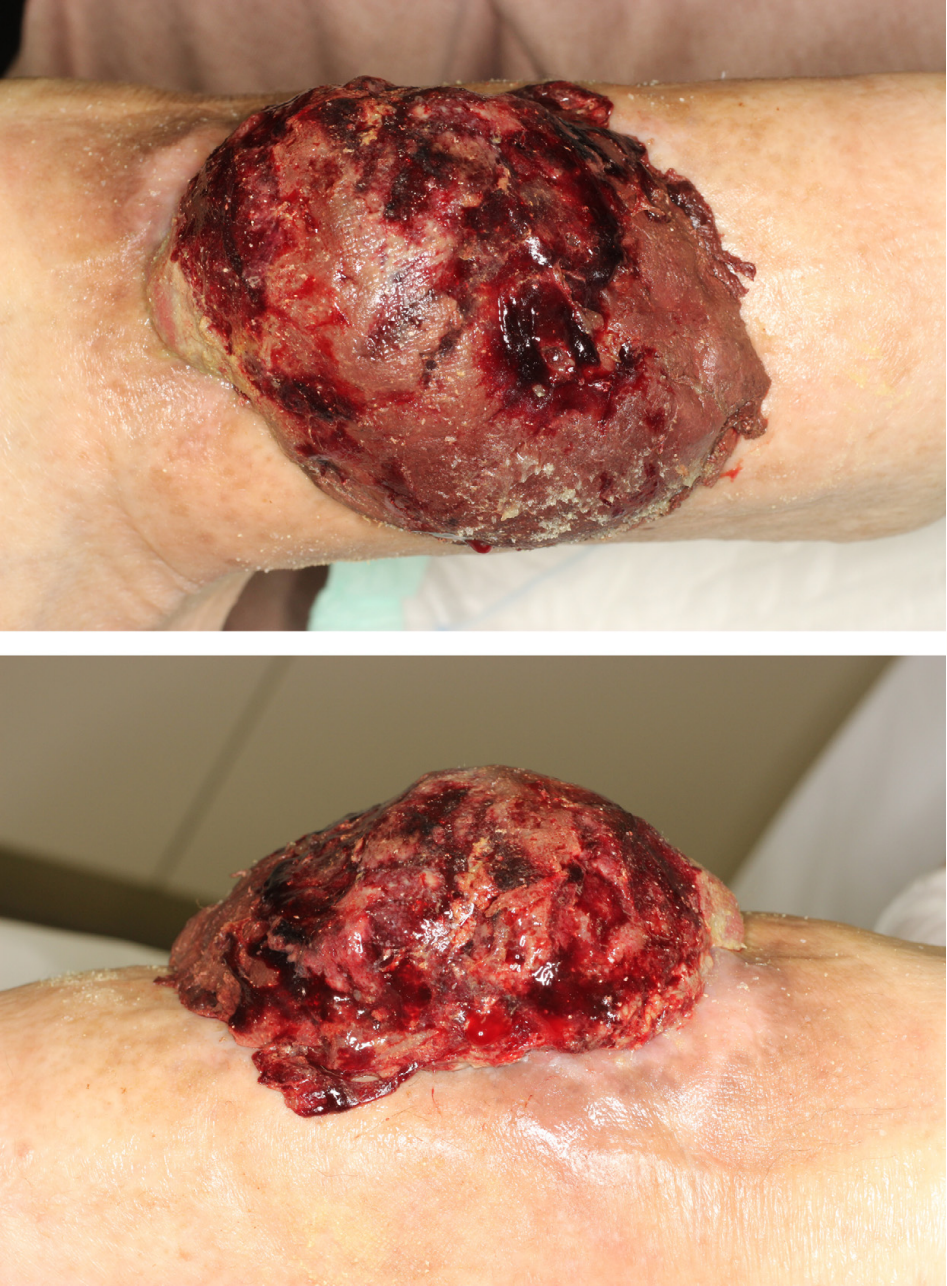
Enlarged hemorrhagic tumor.

A 100‐year‐old woman living in a care facility in a village in Nihonmatsu, Fukushima, was hospitalized due to loss of consciousness. She had experienced fatigue and dizziness with anemia caused by ongoing bleeding from a skin lesion on the right knee. Examination revealed a large (maximum diameter, 20 cm) pedunculated mass on the knee (Fig. 1). The tumor bled easily, and complete resection of the tumor was considered difficult.

**Figure 2:**
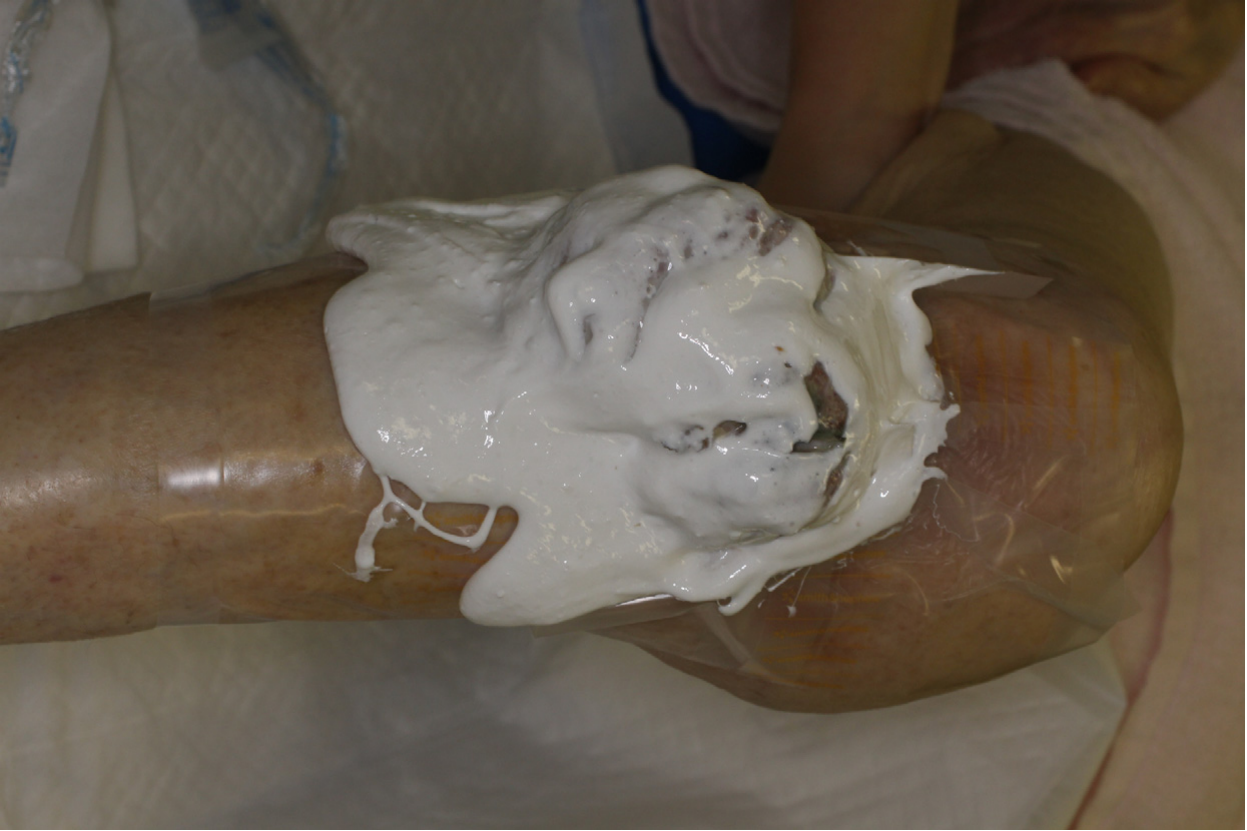
Application of zinc chloride Mohs’ paste to knee lesion.

The mass had been progressively growing for more than 7 years. The cause of the unconsciousness was severe anemia, serious enough to require blood transfusion. For the purpose of hemostasis, zinc chloride Mohs’ paste was applied to the skin lesion (Fig. 2).

**Figure 3:**
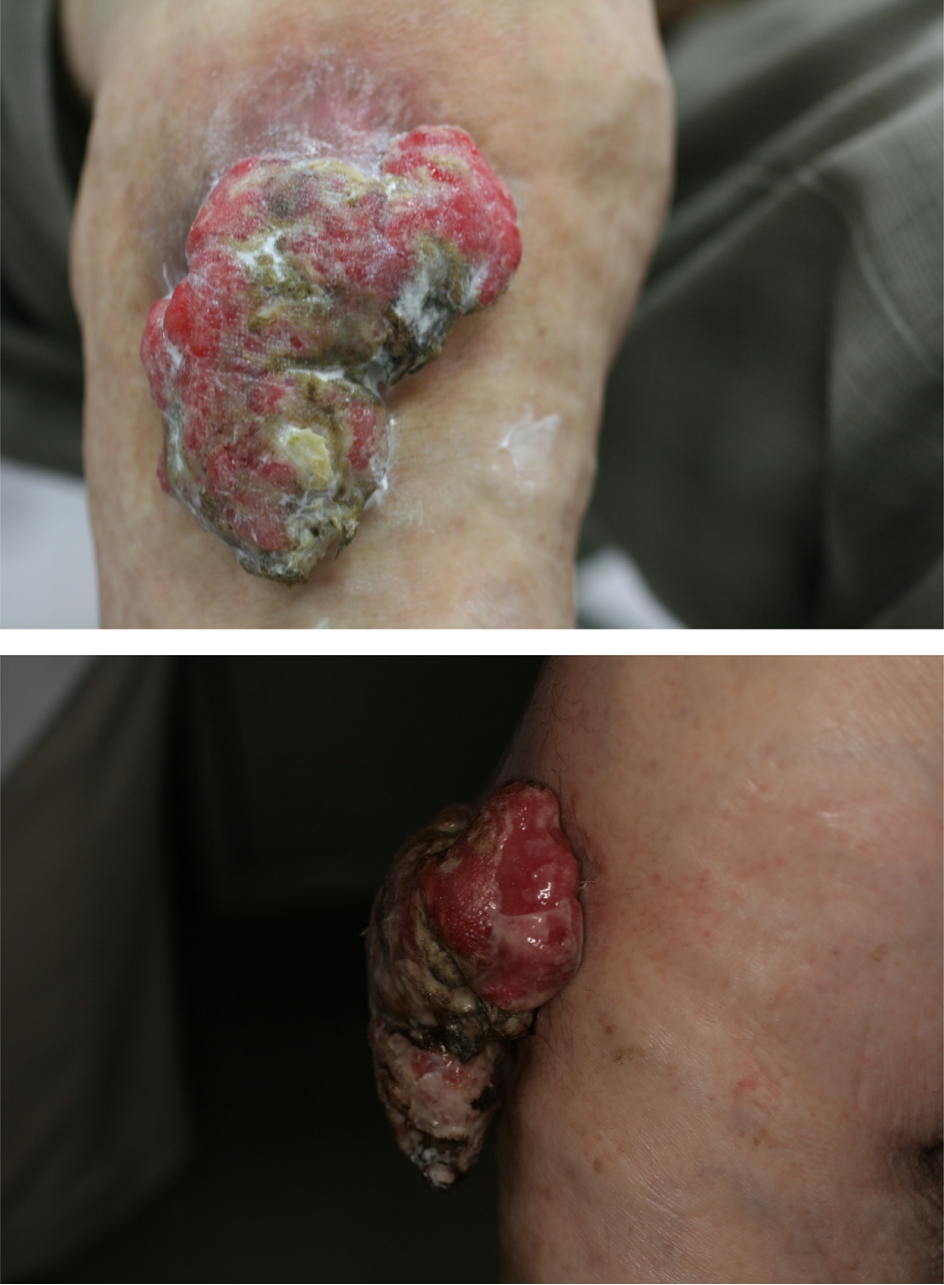
The tumor became smaller.

We continued applying Mohs’ paste every day. The tumor stopped bleeding and became smaller over the course of 2 months (Fig. 3). The tumor was then cauterized and gradually resected.

**Figure 4:**
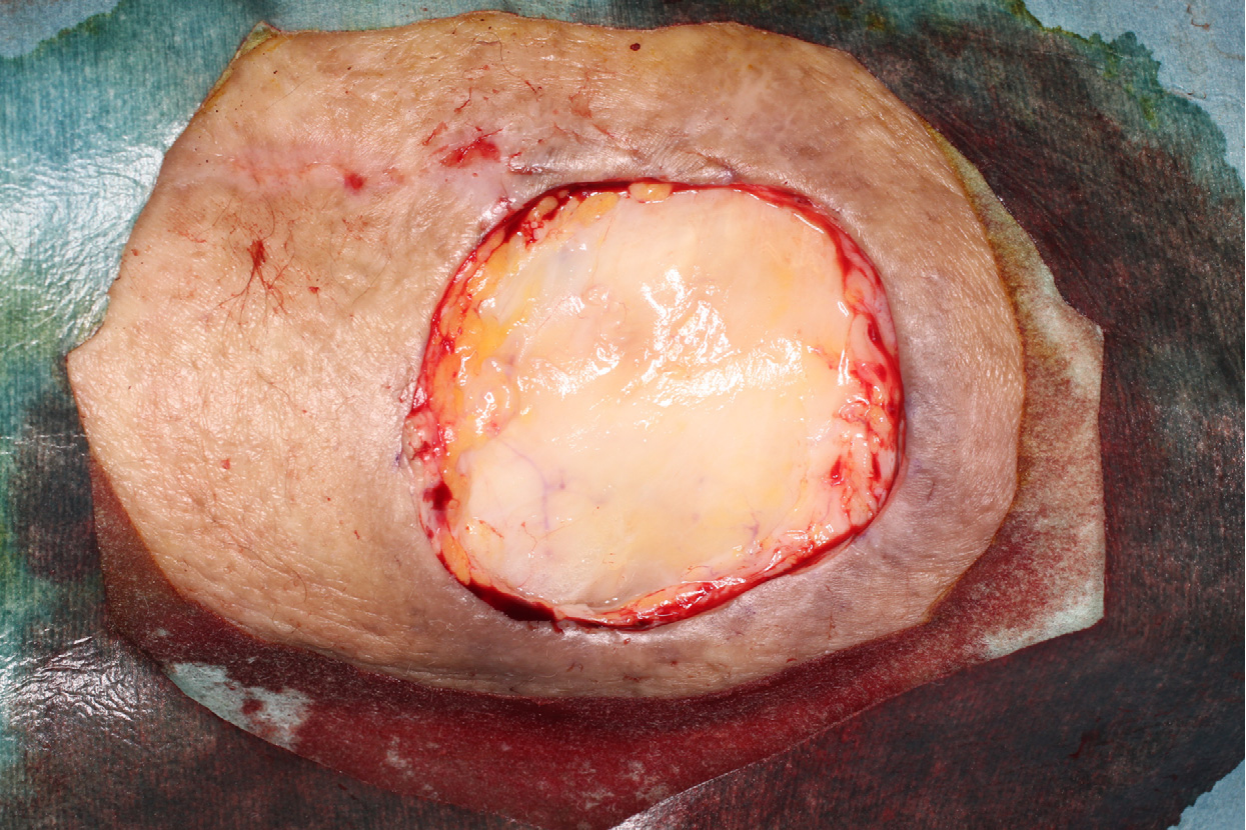
After excision.

**Figure 5:**
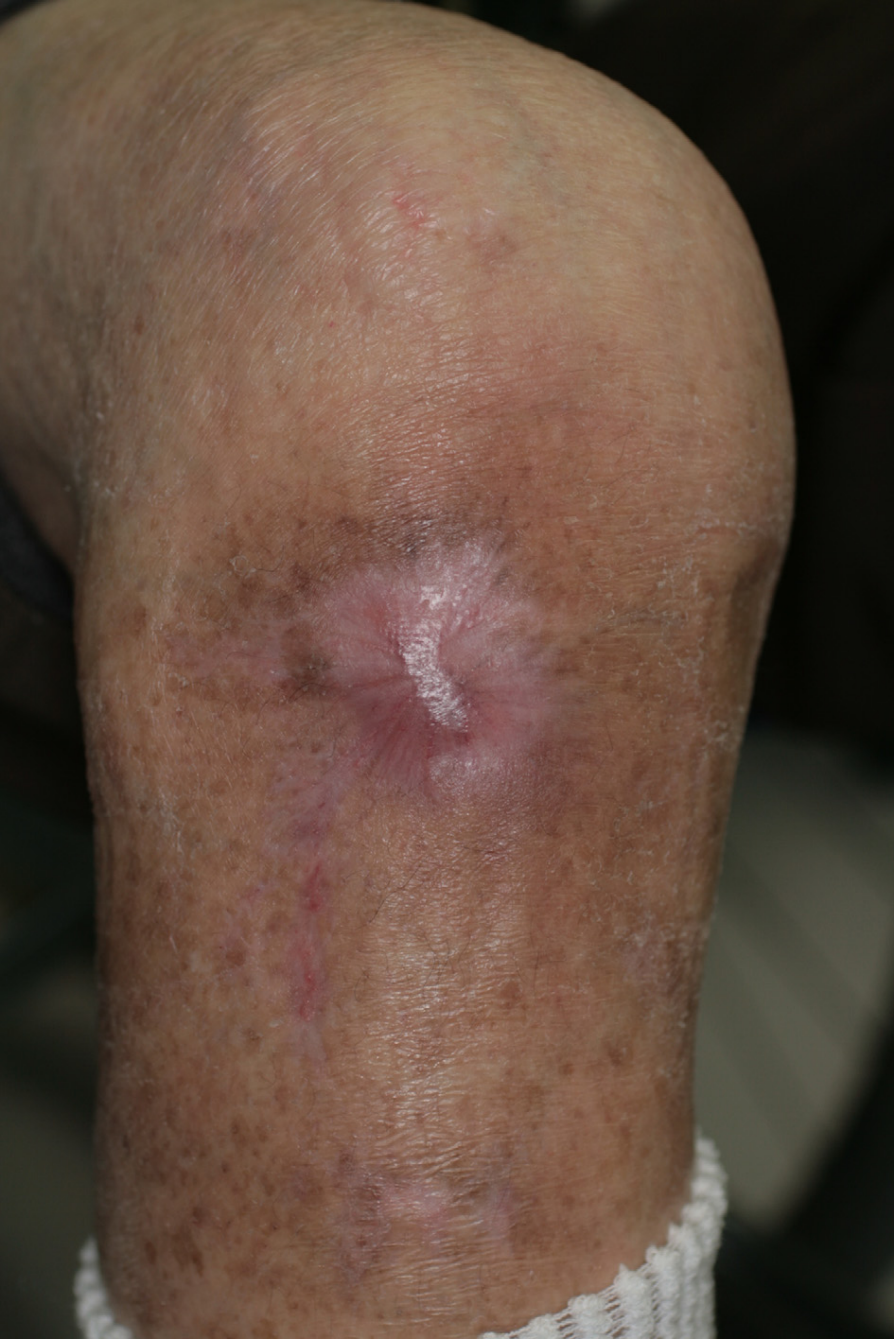
Weeks after reconstruction.

The tumor had a hard stem, and excision was difficult. We therefore removed the lesion surgically using a 5‐mm margin (Fig. 4). Finally, we performed skin autograft. Several weeks later, the only trace of the operation was the scar (Fig. 5). The patient was followed up at 12 months postoperatively, and no local recurrence was noted.

## Discussion

**Figure 6:**
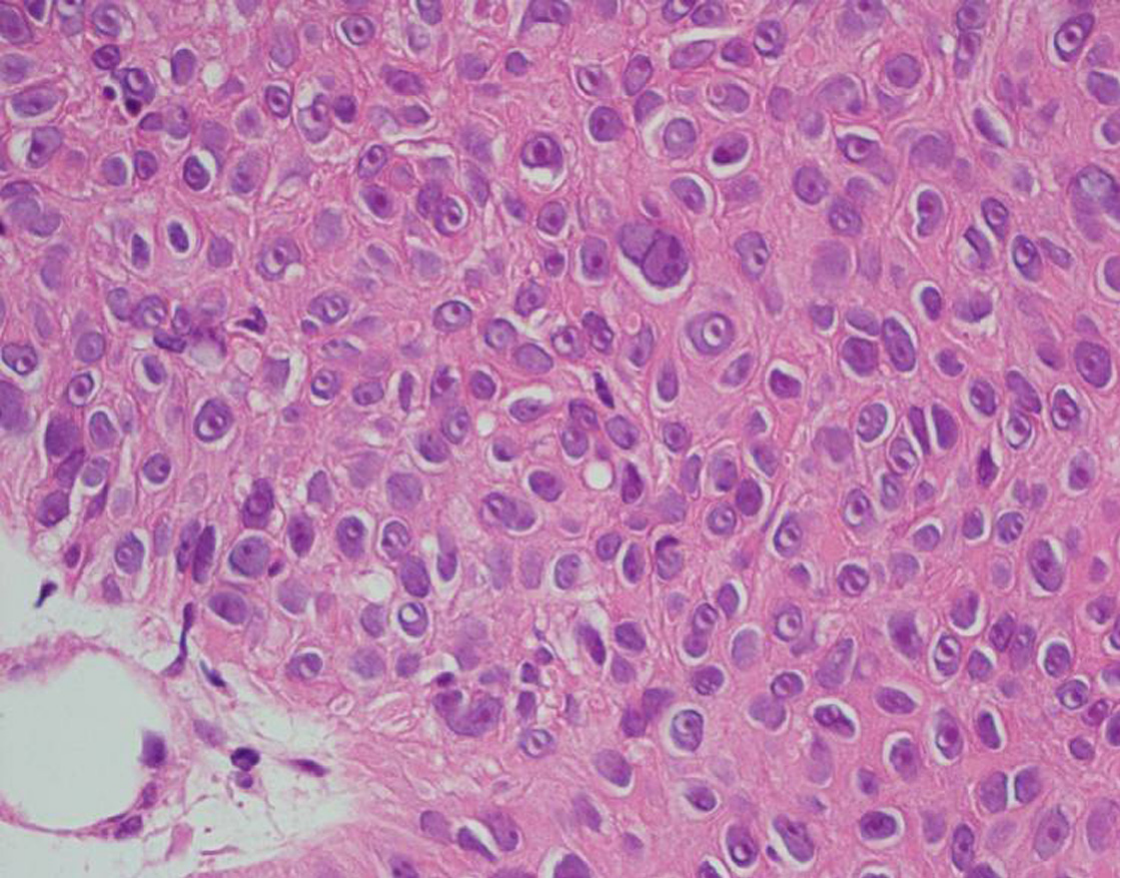
Mixed tumor of the skin Eccrine porocarcinoma with poroid cells, cuticle cells, and prickle cells are apparent in the excision specimen.

The patient had often experienced disturbance of consciousness due to anemia. However, she had been unable to receive adequate treatment in the aftermath of the nuclear power plant leak caused by the 2011 Great East Japan Earthquake and tsunami. Examination revealed a large (20‐cm) pedunculated mass hanging from the knee like the nose of a *tengu*, a Japanese mountain goblin with a very large, red nose. Benign eccrine porocarcinoma arises from within eccrine gland ducts in the epidermis. Malignant transformation is rare, but should be suspected when bleeding occurs 1. Malignant eccrine porocarcinoma was suspected in the present patient due to the malignant histology in specimens from the right knee skin lesion and examination of the completely resected primary tumor. At the time of the first medical examination, she was bleeding from the knee lesion, which was fragile. However, application of Mohs’ paste stopped the bleeding, eventually enabling excision. Because the stem of this pedunculated tumor was hard, we excluded it surgically in an electric scalpel. A surgical margin of 5 mm was applied. On histopathological examination of the excision specimen, cuticle cells and prickle cell cancer were seen to be mixed with eccrine porocarcinoma (Fig. 6). CT revealed no lymph node metastases, but pulmonary metastasis and gallbladder cancer were detected. The tumor was classified as stage IV. Follow‐up at 1 year after excision revealed recurrence on the knee. No chemotherapy was provided after resection, and the patient died of natural causes at 102 years old.

## Authorship

KG: took care of the patient and did the literature search; wrote the preliminary manuscript and patient's case summary: wrote the manuscript and coordinated the project; provided the pathological images; YH: helped in writing the discussion part of the manuscript and made some corrections in the manuscript; provided his specialized and technical help regarding the completion of the manuscript. KG and YR: edited the whole manuscript at the end, checked the accuracy of references, and provided the critical review of the article.

## Conflict of Interest

The authors declare that they have no competing interests.
